# Determination of Water-Soluble Trace Elements in the PM_10_ and PM_2.5_ of Palermo Town (Italy)

**DOI:** 10.3390/ijerph20010724

**Published:** 2022-12-30

**Authors:** Daniela Varrica, Maria Grazia Alaimo

**Affiliations:** Dip. Scienze della Terra e del Mare (DiSTeM), Via Archirafi 22, 90123 Palermo, Italy

**Keywords:** leaching test, water-soluble ions, trace elements, PM_10_ and PM_2.5_, atmospheric pollution

## Abstract

This study contributes to the current knowledge on the solubility of trace elements in the atmospheric particulate matter of the urban area of Palermo. Daily sample filters of PM_10_ and PM_2.5_ were collected in monitoring stations within and outside the urban area, characterized by variable traffic density. The bulk of compositions in PM_10_ and PM_2.5_ were determined by ICP-MS. The water-soluble trace elements (WSTE) and major ion components of particulate matter were determined by ICP-MS and ion chromatography, respectively. A significant difference in the metals content was observed between the samples taken in urban areas and those from suburban areas. The calculated enrichment factor highlights the high values for Cu, Mo, Sb, V, and Zn, confirming the contribution of human activities. The leaching test was applied to PM_10_ and PM_2.5_ filters and showed different behaviors and transport of metals and metalloids. The calculated leaching coefficient highlights the metals typically produced by anthropic activities, compared to those of geogenic origin, are much more soluble in water and have greater mobility. The factor analysis was used to identify the sources of water-soluble ions. The main sources are anthropic, geogenic, and sea spray. The final objective of this study is to obtain, with the aid of leaching experiments on PM_2.5_ and PM_10_ filter samples, information about the bioavailability and mobility of the different metals and metalloids that could be used as the scientific basis for public health intervention and to raise the prevention and control of heavy metal pollution in the urban environment, especially in densely populated areas.

## 1. Introduction

Air pollution is one of the most complex problems facing modern society now on a global level [1]. To date, attention to this phenomenon, based on many scientific studies and many awareness campaigns, has not been indifferent. Starting in 2019, the European Commission proposed a number of policy initiatives with the overall goal of achieving climate neutrality in Europe by 2050 through the European Green Deal [2]. The Zero Pollution Action Plan provides guidance for integrating pollution prevention into all relevant EU policies; it includes targets on air, water, soil, and noise pollution, as well as waste generation and biodiversity [3]. Global environmental pollution, together with the environmental damage it causes, such as the contamination and depletion of many natural resources, is considered a problem due to its adverse effects on human health [4]. Pollutants dispersed in the air, water, and soils come into direct contact with humans, and if ingested or inhaled, they can likely become part of the metabolic cycles causing very serious pathologies as well as genetic mutations [5,6].

Pathologies associated with the atmospheric pollution phenomena are mainly allergies, asthma, autoimmune diseases, diseases of the cardiovascular system and the nervous system, as well as various types of cancer [7]. It is difficult to define a polluted atmosphere based on a standard since it is a constantly evolving reservoir; therefore, air pollution has been recognized as exceeding the limits of the different elements or chemical compounds set by law based on the risks for health and the natural environment [1].

Airborne particulate matter (PM) is considered a complex multi-component mixture generated through a variety of processes and mechanisms and emitted from numerous sources (vehicular traffic, industrial activities, power plants, domestic fuel, resuspension of dust, volcanic emissions, and sea spray aerosols). Anthropogenic sources identified in vehicular traffic are responsible for most of the urban pollution caused by the combustion processes of fossil fuels, which mainly release inorganic gases (SOx, NOx, CO), organic gas (BTX), metals and metalloids, and fine dust (PM) [7]. Exhaust system emissions of metals can arise from the burning of fuels and lubricating oils, and several studies pointed out that, e.g., Ca, Mn, Fe, Cu, Ni, Zn, Cr, and Ba are typical elements from these emissions. Abrasion and wear of components, such as engines, brakes, and tires, result in non-exhaust emissions from vehicles that have been found to be higher sources of Zn, Cu, Fe, Sb, and Ba. Road dust contains, in addition to deposition from the aforementioned sources, elements from soil and abrasion of the road surface (e.g., Ca, Fe, Sr, Si, trace Ti, and Mn) [8].

Furthermore, organic and inorganic secondary particles formed in the atmosphere by chemical processes involving precursor gases emitted from different sources account for a large fraction of PM fine mass [9,10].

Aerosol particles of fine dimensions are recognized as having a strong impact on the environment and having concerning health-related effects. In urban areas, air particle pollution is of particular interest for the possible delayed health effects associated with the continuous exposure to a high-density population [5,11].

A very important characteristic of atmospheric PM is related to particle size as it determines atmospheric life and pulmonary deposition. The danger of the particulate is not only expressed linearly to its quantity or size, but its chemical composition is also of fundamental importance [12,13,14,15,16]. To evaluate pollution and danger levels, information on the mass and total content of trace elements in the particulate is necessary but not sufficient because the effect of trace elements on the environment and humans depends on the form of association in the solid phase to which the elements are related [17]. Toxicological studies have associated the water-soluble fraction of the metal content with a possible harmful component of the particulate matter [18]. The potentially toxic elements of PM according to Directive 2008/50/EC [19] are As, Cd, Cr, Hg, and Pb, which play a crucial role in the generation of reactive oxygen species and therefore in the adverse effects of PM [20,21,22]. To carry out a complete assessment of the potential toxic effects (and therefore the risk to human health) of PM on an area, it is necessary to determine both the elemental composition and the water-soluble fraction. Therefore, the toxicity of the elements and the risks to human health related to them are linked to their bioaccumulation capacity and consequently to their mobility [23].

Through the leaching technique, also known as solid–liquid extraction, which consists of the release of ions or soluble compounds from a solid by means of a solvent, it was possible to observe the ability of the trace elements contained in the PM filters to pass into an aqueous solution.

With the aid of leaching experiments on PM_2.5_ and PM_10_ filter samples, the objective of this study is to obtain information about the bioavailability and mobility of the different metals and metalloids released in an aqueous solution by each sample. This study contributes to current knowledge on the solubility of trace elements in the atmospheric particulate matter of the urban area of Palermo. The study of the release mechanism and speed of mobilization of trace elements, which have a higher concentration in the atmospheric particulate or those which have been leached, can serve as a material on which to base improvements for the evaluation and prediction of the behavior of fine particles in different natural environmental systems.

## 2. Material and Methods

### 2.1. Description of the Study Area

With about 670,000 inhabitants, Palermo is the largest urban area of Sicily, and its metropolitan area is populated by more than one million people. The city (38°06′54.36″ N–13°21′02.88″ E) is situated on the north-western coast of the island along the wide bay “Piana di Palermo”, and it is delimited at the northeast by the Tyrrhenian Sea and is surrounded by mountains (Monti di Palermo) reaching 500–1000 m above sea level. The climate is typically Mediterranean with warm dry summers and moderately rainy winters. The prevailing wind directions are from east and west. The movements of the local air masses are strictly linked to topography. Normally, during the daytime, sea breezes drive the pollutants produced in the city toward the surrounding mountains. During evening and night, mountain breezes take place, which drive the polluted air masses above the city. Potential local pollutants are limited to emissions from vehicular traffic and small manufacturing industries. The study area is entirely covered by sedimentary rocks (limestone, clay, marly-clay, and white or yellow quaternary biocalcarenite) [24].

### 2.2. Sampling Sites

**Figure 1:**
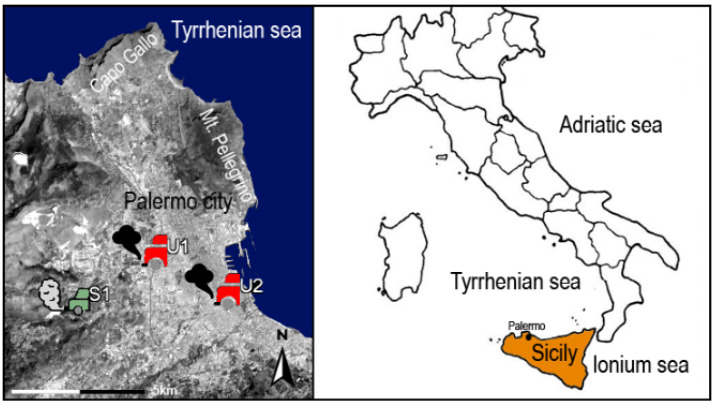
Location of the study area and sampling sites of air-monitoring stations.

A total of 102 daily samples, 62 PM_10_ and 40 PM_2.5_, were collected during the winter months (December–February). To meet the requirements of Directive 1999/30/EC (EU Commission, 1999), PM_10_ sampling was performed according to European Standard EN12341 (CEN, 1998) with a low-volume system equipped with a sampling inlet head (Zambelli Explorer Plus Controller 16) operating at a constant sampling rate (2.3 m^3^h^−1^). Particles were collected on standard 47 mm quartz filters (Advantec, grade QR100). The sampling time was 24 h from midnight to midnight. Similarly, PM_2.5_ sampling was performed according to European standard EN 14907 (CEN, 2005). The initial and final weighings of PM_10_ and PM_2.5_ filters were carried out in a temperature- and humidity-controlled room (T = 20 ± 1 °C, RH = 50 ± 5%) after the filters had been conditioned for 48 h before and after sampling. Three air-quality-monitoring stations belonging to the municipal monitoring network (RAP-ex AMIA) were chosen for this study (Figure 1). The urban station PM_2.5_ (U1) is located close to a crossroads with traffic lights at pedestrian crossings and is characterized by high-traffic flow, consisting of cars, heavy-duty vehicles, and buses. The urban station PM_10_ (U2) is situated in a large square in front of the railway station, exposed to heavy traffic composed of cars as well as urban and regional buses. The suburban station PM_10_ (S1) is a background station situated leeward of the sea breeze without any direct influence from urban activities. It has a lower traffic density than the other stations and was selected as a control site to monitor the hypothetical background level of pollution.

### 2.3. Analytical Procedures

The PM_2.5_ and PM_10_ samples were analyzed for insoluble ions. To determine water-soluble ions, filters were placed in vials and ultrasonicated at room temperature for 24 h in 20 mL ultrapure 18 MΩ water. The extracts were filtered through a 0.45 μm pore size polytetrafluoroethylene filter (Sartorius) and then stored in sterile 50 mL polypropylene centrifuge tubes. The pH, EC, and redox potential of the solution in the water extraction step were measured on all samples in an aliquot of the solution extract. pH measurements were carried out using a glass electrode connected to a VWR pH100-m after calibration with pH 4.01 and 7.00 buffer solutions. The electrical conductivity was measured by a VWR EC300 instrument, previously calibrated by the conductivity solution HI 70031 (HANNA). EC values are reported at 25 °C. A SenTix ORP combination Pt electrode, calibrated daily against ZoBell’s solution [25], was employed to measure redox potentials.

The remaining part was acidified to 2% HNO_3_ to prevent metal adsorption and stored at 4 °C for later analysis. Concentrated HNO_3_, HClO_4_, and HF acids were then added to the filters to give a 5 mL total volume of 3:1:1 *v*/*v* acid digested in a microwave. After digestion, the solutions were diluted by the addition of 18 MΩ cm deionized water to reach a volume of 50 mL.

Water-soluble ions were analyzed the day after the extraction procedure for Ca^2+^, Mg^2+^, Na^+^, K^+^, Cl^−^, SO_4_^2–^, and NO_3_^–^ ions by ion chromatography (Dionex 100), and NH_4_^+^ ions by spectrophotometer UV-Vis using Nessler’s reagent at λ = 420 nm (Thermo Scientific Evolution 600).

The limit of detection was evaluated by solution extracts for three blank filters in 0.03–0.07 and 0.03–0.05 mg L^−1^ for cations and anions, respectively.

Eighteen trace elements (Al, As, Ba, Co, Cr, Cu, Fe, Li, Mn, Mo, Ni, Pb, Rb, Sb, Sr, U, V, Zn) were measured by inductively coupled mass spectrometry (ICP-MS, Perkin-Elmer, Elan 6100 DRC-e, SD, CA, USA) after addition of Re-Sc-Y as internal standards on a total of 102 filter samples (40 at U1, 40 at U2, 22 at S1, respectively) both for the determination of the total content and water-soluble trace elements. For As, Cr, Fe, and V the ICP-MS was operated in DRC mode with CH_4_ as the reaction gas. All standard solutions were prepared with ultra-pure deionized water, the ICP Multi Element Standard Solutions XXI CertiPUR, and the Mo and Sb CertiPUR standards (MERCK). In the bulk samples, to minimize the matrix effects, the standard addition technique was used for all the metal determinations; the analytical precision, estimated by carrying out several replicates, was in the range of 1–10% for all the analyzed elements, except for As and Cr, which resulted in 15%. The validity of the whole analytical procedure was checked using the NIST standard reference material Road Dust, SRM 1648. The metal recovery rates of certified elements in the reference material were between 84% and 95%, with an average value of 90%. In the water-soluble trace elements, the precision of the analytical results was estimated by running triplicate analyses every tenth sample and fell within the range of 3–12%. Accuracy (±10%) was assessed by running SRM-1640 (groundwater) and TMRAIN-95 (rainwater) reference standard materials. The limit of detection (LOD) and the limit of quantitation (LOQ) were evaluated by solution extracts from three blank filters for each element in Al (0.07 μg L^−1^; 0.12 μg L^−1^), As (0.02 μg L^−1^; 0.04 μg L^−1^), Ba (0.04 μg L^−1^; 0.08 μg L^−1^), Co (0.03 μg L^−1^; 0.08 μg L^−1^), Cr (0.04 μg L^−1^; 0.11 μg L^−1^), Cu (0.04 μg L^−1^; 0.08 μg L^−1^), Fe (0.04 μg L^−1^; 0.11 μg L^−1^), Li (0.01 μg L^−1^; 0.04 μg L^−1^), Mn (0.01 μg L^−1^; 0.02 μg L^−1^), Mo (0.05 μg L^−1^; 0.10 μg L^−1^), Ni (0.04 μg L^−1^; 0.07 μg L^−1^), Pb (0.04 μg L^−1^; 0.08 μg L^−1^), Rb (0.01 μg L^−1^; 0.02 μg L^−1^), Sb (0.004 μg L^−1^; 0.009 μg L^−1^), Sr (0.01 μg L^−1^; 0.04 μg L^−1^), U (0.001 μg L^−1^; 0.002 μg L^−1^), V (0.05 μg L^−1^; 0.08 μg L^−1^), Zn (0.05 μg L^−1^; 0.10 μg L^−1^), respectively. Analyses were carried out at Dept. Scienze della Terra e del Mare, University of Palermo.

### 2.4. Statistical Analysis

Data were analyzed statistically by the STATISTICA program (Tulsa, OK, USA), Stat-Soft version 6.0. All the tests in this study were considered significant at *p* < 0.05. The Shapiro–Wilk test with a level of significance set at *p* < 0.05 was used to verify the normality of data distribution. The non-parametric Mann–Whitney test at *p* < 0.05 was also used to verify the statistical significance of observed differences between PM_10_ filters.

## 3. Results and Discussion

### 3.1. Total Mass and Trace Elements

A summary of the total PM_10_ and PM_2.5_ mass concentrations for the urban and peripheral stations is given in Table 1.

The highest mean PM_10_ value was observed at the urban site (35 μg m^−3^), one of the most heavily traffic-exposed sites in the study area, followed by the peripheral station (16 μg m^−3^). In addition, the high vehicular impact on urban pollution is confirmed by the PM_2.5_ atmospheric particulate content, which reports an average value of 29 μg m^−3^. The values measured in Palermo in this study show a situation comparable to other Italian and European cities [26].

**Figure 2:**
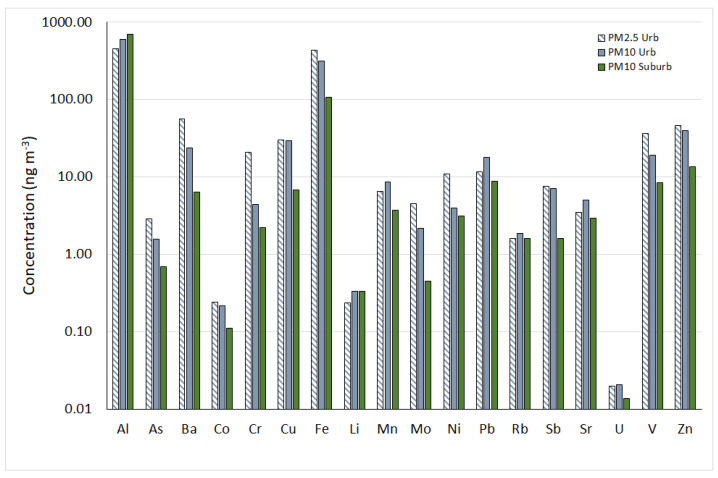
Average distribution of trace element concentrations in PM_2.5_ and PM_10_. Data are expressed in ng m^−3^.

The average distribution of elemental concentrations in PM_2.5_ and PM_10_ is shown in Figure 2.

The metal and metalloid profiles have similar patterns between the two fractions. In general, the abundance of the trace elements in urban sites with a decreasing trend in PM_2.5_ was: Al, Fe > Ba > Zn > V > Cu > Cr > Pb, Ni > Sb > Mn > Mo > Sr > As > Rb > Li, Co > U; whereas the trend in PM_10_ was: Al > Fe > Zn > Cu, Ba > Pb, V > Mn, Sb > Sr, Cr > Ni > Mo > Rb > As > Li > Co > U. At the suburban station, the order of abundance for PM_10_ was Al > Fe > Zn > Pb, V > Cu, Ba > Mn, Ni, Sr > Cr > Sb, Rb > As > Mo, Li > Co > U.

The metals with the greatest concentrations across all sites were Al and Fe, accounting for about 70–80% of the total trace elements, indicating the significant contribution of soil and resuspended mineral dust to atmospheric PM_2.5_ and PM_10_. Other elements, such as As, Ba, Cr, Mo, Ni, and V, had higher concentrations in the PM_2.5_ fraction than PM_10_, confirming the role of anthropic processes, such as the mechanical abrasion release of the metal structures of vehicles, engine components, tires, and brake linings.

#### Enrichment Factor

**Figure 3:**
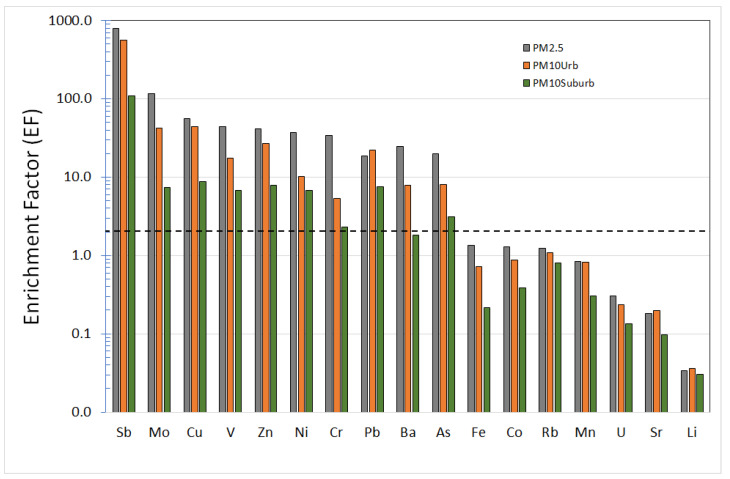
Average enrichment factors (EFs) for the analyzed elements in PM_2.5_ and PM_10_ samples. The dashed line indicates the boundary between enriched and non-enriched.

To assess the contribution of anthropogenic emissions to atmospheric element levels within the urban area, the enrichment factor (EF) was computed as the ratio of the concentration of each element in PM samples to its average abundance in local soils. According to Varrica et al. [27], the average local soil (LS) for the studied area is considered to be made up of carbonate rocks (80%), clay minerals (10%), and “Terra Rossa” soil (10%). Aluminum was selected as a reference element. The mean EFs estimated for each element are shown in Figure 3. Based on Hernandez et al.’s considerations [28], EF values ranging between 0.5 and 2 can be considered in the range of natural variability, whereas ratios greater than 2 indicate some enrichment corresponding mainly to anthropogenic inputs.

Elements, such as Co, Fe, Li, Mn, Sr, and U, as they are known, are predominantly dominated by a crustal origin and are not enriched in relation to the local soil (LS) with an EF < 1. The figure shows some elements are particularly enriched in the fine fraction 2.5 compared to the coarse fraction. Elements, such as As, Ba, Cr, and Ni, have an enrichment with EF < 10 in PM_10_ samples compared to PM_2.5_ samples which have an EF > 10. The enrichment factor calculated for lead continues to confirm the presence of this element in atmospheric particulate matter. Cu, Mo, Sb, V, and Zn have an EF > 20 in both fractions. Particular attention should be paid to the enrichment factors of antimony with EFs calculated above 500 for both PM_10_ and PM_2.5_. The higher concentrations of these elements in urban PM reveal the fundamental contribution of human activities. The lowest EFs were observed in the suburban site for all elements analyzed.

Antimony, copper, and molybdenum are elements identified with vehicular traffic pollution released by brake wear and linings in urban environments. Their concentrations over time in urban environments have grown to impose on identifying elements of vehicular traffic. The Cu/Sb ratio in particular is used as a fingerprinting tool in identifying the contribution of road vehicles to traffic-derived PM [29,30]. Sternbeck et al. [31] proposed a typical Cu/Sb ratio of 4.6 ± 2.3, resulting from brake and lining wear release. In this study, the ratio varied between 3.9 and 4.1 at urban PM_2.5_ and PM_10_ sites, respectively. The source of molybdenum in an urban area is related to vehicle brake wear, which can contribute up to 50% of PM_10_ and 12% of PM_2.5_ emissions, respectively [32].

### 3.2. Leaching Test

The water-soluble trace element (WSTE) components of PM are one of the main factors responsible for PM-induced toxicity [33]. Table 2 shows the data relating to the main chemical–physical parameters measured in the leaching filters. The measured conductivity values reflect the quantities of material deposited on the filter. The measured redox potentials are similar in both PM_2.5_ and PM_10_ samples, while the PM_2.5_ filters show a slightly lower pH value than the PM_10_ filters. In all phases of the experiment, pH trend shows a tendency towards neutrality. The presence of carbonate minerals in atmospheric PM can become the main neutralizing agent [34]. Studies carried out on the pH of the rainwater in the study area have shown processes that contribute to the neutralization of rain are attributable to the contribution of marine sprays and the dissolution of carbonate rocks [35].

The leachability of heavy metals bound to atmospheric particles is strongly dependent upon chemical speciation. Since atmospheric particles at a given site generally come from different sources with varying mineralogical compositions, the contributions related to the leaching of trace elements are expected to vary between sites [36]. Table 3 shows the concentration of WSTE and major ions in the PM_2.5_ and PM_10_ samples.

The data indicate large fractions of total PM_10_ in urban and suburban sites (39–45% in weight, respectively) are made up of water-soluble major ions, while the soluble component in fine fraction PM_2.5_ is reduced to 27% in weight. The order of abundance of ionic concentrations expressed in μg m^−3^ shows a prevalence of the NO_3_^−^ ion for anions followed by the SO_4_^2−^ ion in urban filters of both PM_10_ and PM_2.5_. In the peripheral station, the sulfate ion is more abundant, followed by the nitrate ion. The presence of sulfate ions in the lower atmosphere is generally assumed to be a secondary airborne particulate formed from the gas-to-particle conversion of SO_2_. Nitrate ions are derived from the reaction of hydroxyl radicals formed by the photolysis of ozone molecules with NO_x_ emitted by fossil fuel combustion. The concentrations of NO_3_^−^, SO_4_^2−^, and NH_4_^+^ in urban environments demonstrate the presence of secondary origin particulate matter, which is formed as a result of reactions in the atmosphere between acid gases and ammonia, according to a sequence of neutralization reactions [34,37,38]. Across all sites, the most abundant cation is the Ca^2+^ ion, followed by the NH_4_^+^ ion in the urban stations, and the Na^+^ ion in the suburban site. The presence of Na^+^, Cl^−^, and K^+^ ions in the atmospheric particulate matter indicates a geogenic origin to be identified almost exclusively from the marine spray. The ion concentrations of Ca^2+^ and Mg^2+^ in the atmospheric particulate matter may derive from the alteration of carbonate rocks present in the study area, even if, for the Mg, it is not possible to exclude an identifiable component in the sea spray.

The concentration of the analyzed metals and metalloids follows the orders of abundance:

PM_2.5_: Fe > Zn > Ba > Cu > Sr > Mn > V > Sb > Al > Mo > Pb > Cr > Rb > Ni > As > Li > Co > U

PM_10Urb_: Fe > Zn > Ba > Cu > Sr > V > Mn > Sb > Al > Cr > Pb > Mo > Rb > Ni > As > Li > Co > U

PM_10Suburb_: Zn > Fe > Ba > Cu > Sr > V > Mn > Al > Pb > Sb > Mo > Cr > Ni > Rb > As > Li > Co > U

The differences are most evident between the suburban and the urban stations. The distributions between urban stations (PM_10_ and PM_2.5_) are similar. Some variation in the order of abundance between Mn-V and Cr-Mo-Pb is observed. By comparing the concentrations of trace elements present in the leached PM_10_ of urban and extra-urban stations with a non-parametric Mann–Whitney test (*p* < 0.05), the result is differences observed for some elements, such as Ba, Cu, Fe, Pb, and Sb, are statistically significant.

#### 3.2.1. Leaching Coefficient R_ls_

**Figure 4:**
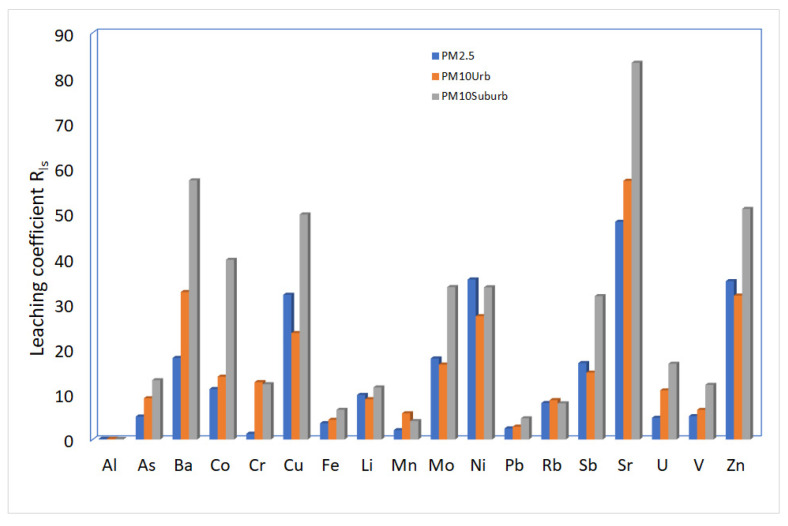
Distribution leaching coefficient, R_ls_, for metals and metalloids in each of the PM_10_ and PM_2.5_ size fractions.

In this study, to evaluate the behavior and transport of the analyzed metals and metalloids, it was considered appropriate to calculate the leaching coefficient, R_ls_, as the ratio between the water-soluble metal and the elemental concentration associated with each of the size fractions PM_10_ and PM_2.5_. The leaching coefficient, R_ls_, is a significant parameter used to describe the distribution of a species between a solid and aqueous matrix after equilibrium. Figure 4 shows the leaching coefficient of the R_ls_ distribution for metals and metalloids in the PM_10_ and PM_2.5_ size fractions.

For PM_10_, the R_ls_ index was <10% for Al, Pb, Mn, V, and As and >40% for Sr. We found elements, such as Sb, Mo, Ni, Cu, Zn, and Ba, in the range of 15–35%, which is considered to be water soluble. In the PM_2.5_ size fraction, the R_ls_ index < 10% was similar to the PM_10_ fraction for Al, As, Co, Li, Mn, Pb, Rb, and V. For Zn, Cu, Mo, Ni, and Sb, the R_ls_ index in PM_2.5_ was higher than the PM_10_ size fractions. The element that enjoys a lower solubility is aluminum with percentages equal in the leached PM_2.5_ and PM_10_. Aluminum of a crustal origin is generally present in silicate minerals, which in a water solution with neutral pH show low solubility. From the figure, it appears the elements that have a medium-higher solubility are Ba, Cu, Mo, Ni, Sb, Sr, and Zn. The origin of these elements in atmospheric particulate matter is exclusively anthropic with the exception of strontium and barium. The high rate of leaching found in Sr and Ba is mainly linked to their crustal origin as they are present in the carbonate rocks surrounding the study area.

In general, the different solubility may depend on chemical bonds, the size of the particulate, and its origin. According to the figure, the fine fraction shows a more significant proportion of water-soluble elements for those elements of typical anthropogenic origin. In contrast, some terrigenous metals are more soluble in the PM_10_ fraction.

Some elements, such as Ni, Sb, and Zn, adversely affect human health. In general, the solubility of nickel in the atmospheric particulate of geogenic origin is linked to silicate minerals, which are not very soluble in water, while anthropogenic nickel compounds are mainly soluble species, such as nickel sulfate [39]. In recent years, several studies have been performed on antimony speciation as Sb(III) has been classified by AIRC as probably being carcinogenic to humans. Some speciation studies on solids have verified in the atmospheric particulate matter of both PM_2.5_ and PM_10_, antimony is mainly present as Sb(V) or mixed layers between Sb(III) and Sb(V) [40]. Several studies on the solubility of antimony have verified at high concentrations, the prevailing form is Sb(V) [41].

Although zinc is considered to be a metal with low environmental mobility [17], our study shows it is among the most soluble elements, which is in agreement with what was also observed by Manousakas et al. [42]. From these considerations, it is possible to deduce zinc is found in urban environments in the form of the soluble salt ZnSO_4_.

In general, it is possible to note the metals typically produced by anthropic activities, compared to those of geogenic origin, are much more soluble in water and have greater mobility. This is because the metal present in the atmospheric particulate is mainly derived from anthropic processes of abrasion of metal parts or condensing particles of hot vapor or metals that have condensed on the surface of other particles and therefore tend to be more labile than metal bound within the crustal material [18].

#### 3.2.2. Factor Analysis

To identify the sources of water-soluble ions in the particulate, we used factor analysis (FA). The input variables were concentrations of 11 selected elements in the filter samples. The load factors of the calculated raw factors were rotated by Kaiser’s varimax rotation scheme [43].

The factorial loads obtained for the three-factor model are shown in Table 4. A factor analysis indicates approximately 72% of the variance can be explained by the first three factors. Factor 1, which shows high positive loads on Cr, Cu, Sb, and Zn elements, represents 31% of the total variance in the database. These are typical elements associated with anthropogenic factors, identified with non-exhaust vehicle emissions. The dominant elements in Factor 2 are Ca, Li, and Sr (23% of the total variance). This factor is mainly attributed to the influence of the geogenic source. The Factor 3 profile, which represents an additional 17% of the residual variance, is determined by elements such as Cl and Na. This factor is representative of the important contribution of sea spray aerosol to PM concentrations. To estimate how much sea spray contributed to the total mass of PM_2.5_ and PM_10_, the sea-salt concentration was calculated from a water-soluble Na and seawater composition [44], assuming the total marine origin of Na. The results underline a higher contribution in coarse particulate filters (24%) than in fine particulate filters (16%), with higher percentages in the suburban station.

## 4. Conclusions

Trace element concentrations of PM_2.5_ and PM_10_ measured in the Palermo town highlight the metal and metalloid profiles have similar patterns between the two fractions. A significant difference in trace element contents was observed between the samples taken in an urban area and those from a suburban area. The metals with the greatest concentrations across all sites were Al and Fe, accounting for about 70–80% of the total trace elements, indicating the significant contribution of soil and resuspended mineral dust. Other elements, such as As, Ba, Cr, Mo, Ni, and V, had higher concentrations in the PM_2.5_ fraction than PM_10_, confirming the anthropic emissions. The enrichment factor (EF) calculated highlights the anthropogenic contribution in the study area for Cu, Mo, Sb, V, and Zn (EF > 20). The results obtained from the leaching test in two PM fractions show the different behaviors and transport of metals and metalloids. The leaching index (R_ls_) calculated for metals and metalloids highlights the elements with R_ls_ index > 35% (Ba, Cu, Mo, Ni, Sb, Sr, and Zn) are exclusively anthropic sources except for Ba and Sr, which are linked to crustal origin. The sources of water-soluble ions in the particulate matter were identified by factor analysis (FA), which lead us to conclude Cr, Cu, Sb, and Zn are typical elements linked to non-exhaust vehicle emissions; Ca, Li, and Sr are identified to a geogenic source; Cl and Na represent an important contribution of sea spray to PM concentrations.

In general, it is possible to note the metals typically produced by anthropic activities, compared to those of geogenic origin, are much more soluble in water and have greater mobility as the metal present in the atmospheric particulate is mainly derived from anthropic processes. The affinity of metals for the individual phases of the mineralogical species to which they are associated and their possible origin may constitute a valid aid for the bodies responsible for implementing programs and measures to be taken for the protection of public health. This study demonstrates the determination of water-soluble trace elements in PM_2.5_ and PM_10_ is a very important tool for implementing knowledge on the mobility, bioavailability, and toxicity of trace elements depending on the chemical forms in which they occur. Together with the determination of the total concentrations of trace elements, it provides an in-depth analysis to evaluate the level of pollution in the urban environment, providing insights into developing policies for strategic management aimed at the prevention and control of heavy metal pollution in the urban environment, especially in densely populated areas.

## Figures and Tables

**Table 1:** Characteristics of PM_10_ and PM_2.5_ samples at the three monitoring stations. Mass -concentrations expressed in μg m^−3^. The number of samples for each monitoring station is shown in brackets.

	PM_2.5_	PM_10_	
	Urban (40)	Urban (40)	Suburban (22)
Mean	29	35	16
Std.Dev.	4	11	7
Min	20	16	8
Max	31	69	44

**Table 2:** Chemical–physical parameters measured in the leaching filters. EC: electrical conductivity (μS/cm), E_H_ (mV).

		pH	EC	E_H_
			μS cm^−1^	mV
Urban station TPM_2.5_	Mean	6.5	15	256
	Std.Dev.	0.2	15	263
	Min	6.1	0.5	209
	Max	6.8	47	289
Urban station TPM_10_	Mean	6.5	16	246
	Std.Dev.	0.2	15	263
	Min	6.1	0.5	209
	Max	6.8	47	289
Suburban station TPM_10_	Mean	6.7	10	249
	Std.Dev.	0.2	1.1	20
	Min	6.5	9	213
	Max	6.9	12	274

**Table 3:** Concentrations of water-soluble trace elements (WSTE) and the major ions in PM_2.5_ and PM_10_ samples. Data are expressed in ng m^−3^ for trace elements and μg m^−3^ for major-ions. Test U: Mann–Whitney test (*p* < 0.05). The level of statistical significance is indicated in italics.

	PM_2.5_		PM_10_Urb		PM_10_Suburb		Test U_PM10Urb-Suburb_
	Mean	Median	Mean	Median	Mean	Median	*p*-Level
**Al**	0.79	0.50	1.00	0.47	0.92	0.75	0.1097
**As**	0.15	0.13	0.14	0.13	0.13	0.14	0.8227
**Ba**	8.04	7.16	7.70	6.61	3.65	4.20	*0.0024*
**Co**	0.04	0.04	0.04	0.04	0.04	0.05	0.2802
**Cr**	0.27	0.25	0.57	0.37	0.27	0.29	0.0698
**Cu**	5.88	5.28	4.86	4.21	3.35	3.36	*0.0207*
**Fe**	15.8	14.0	13.7	13.0	7.09	7.62	*0.0092*
**Li**	0.04	0.03	0.05	0.05	0.04	0.04	0.2172
**Mn**	2.33	2.30	2.37	2.11	1.60	1.72	0.3036
**Mo**	0.52	0.50	0.39	0.37	0.25	0.21	0.5326
**Ni**	0.22	0.20	0.23	0.21	0.25	0.21	0.7086
**Pb**	0.28	0.22	0.54	0.36	1.41	1.26	*0.0029*
**Rb**	0.13	0.12	0.16	0.15	0.23	0.23	0.6718
**Sb**	1.28	1.09	1.01	0.89	0.51	0.35	*0.0129*
**Sr**	1.70	1.57	2.91	2.65	2.45	2.38	0.1811
**U**	0.002	0.001	0.004	0.002	0.002	0.001	0.7565
**V**	1.87	1.50	1.25	1.04	1.73	1.82	0.0984
**Zn**	9.13	8.71	11.8	9.92	13.3	9.42	0.9801
**Ca^2+^**	1.97	2.07	1.74	1.43	1.36	1.12	0.2788
**Cl^−^**	0.47	0.08	1.88	1.47	0.12	0.10	*0.0004*
**K^+^**	0.23	0.22	0.88	0.24	1.21	1.16	0.6144
**Mg^2+^**	0.17	0.14	0.59	0.30	0.64	0.49	*0.0014*
**Na^+^**	0.61	0.35	1.27	1.27	0.92	0.64	*0.0001*
**NH_4_^+^**	0.81	0.55	1.24	0.60	0.71	0.65	0.8012
**NO_3_^−^**	2.06	0.85	3.87	1.72	0.82	0.85	*0.0008*
**SO_4_^2−^**	1.45	1.37	2.15	1.59	1.51	1.19	0.1990

**Table 4:** Factor loadings (Varimax rotation) for the filter samples of PM_10_ and PM_2.5_ (*p* < 0.05).

	Factor 1	Factor 2	Factor 3
Ca	−0.42	0.06	0.65
Cl	−0.04	0.92	0.09
Cr	0.80	0.12	0.13
Cu	0.74	0.00	0.60
Li	0.07	−0.22	0.71
Mo	−0.29	0.06	0.07
Na	−0.04	0.95	−0.07
Sb	0.60	0.08	0.21
SO_4_	0.12	0.07	0.50
Sr	0.33	0.11	0.75
Zn	0.76	−0.21	0.11
Expl.Var	2.53	1.88	2.18
Prp.Totl	0.31	0.17	0.24

## Data Availability

The data presented in this study are available on request from the corresponding author.
